# 

**Published:** 1881-11

**Authors:** 


### CONTENTS:

ART. I.—Antiseptic Treatment of Alveolar Abscesses. By Dr. W. A. Pease.......... 289
ART. II.—Treatment of Teeth with Dead or Dying Pulps, and also of Alveolar Abscess. By Dr. H. H. Townsend.................... 302
ART. III.—Review of "Unfortunate Interference."........................ 318
ART. IV.—Odontalgia. By Ed. H. Bowne, D. D. S........................ 319
ART. V.—Proceedings of Southern Dental Association. (Continued)............ 321
ART. VI.—Concerning a So-Called Dental College............................ 329

### EDITORIAL, ETC.

Proceedings of Southern Dental Association. Remarks on........................ 330
Tin Foil.................................................. 331
Dr. Oliver's Journal.......................................... 331

### OBITUARY.

Professor E. Lloyd Howard, M. D.................................. 332

### MONTHLY SUMMARY.

Women as Physicians.......................................... 335
Death from Nitrous Oxide...................................... 335

